# Direct Quantification of Cell-Free, Circulating DNA from Unpurified Plasma

**DOI:** 10.1371/journal.pone.0087838

**Published:** 2014-03-03

**Authors:** Sarah Breitbach, Suzan Tug, Susanne Helmig, Daniela Zahn, Thomas Kubiak, Matthias Michal, Tommaso Gori, Tobias Ehlert, Thomas Beiter, Perikles Simon

**Affiliations:** 1 Department of Sports Medicine, Rehabilitation and Prevention, Johannes Gutenberg-University of Mainz, Mainz, Germany; 2 Department of Health Psychology, Johannes Gutenberg-University of Mainz, Mainz, Germany; 3 Department of Psychosomatic Medicine and Psychotherapy, Johannes Gutenberg-University of Mainz, Mainz, Germany; 4 Department of Cardiology, Angiology and Internal Medicine, Johannes Gutenberg-University of Mainz, Mainz, Germany; 5 Department of Sports Medicine, Medical Clinic, Eberhard-Karls-University of Tuebingen, Tuebingen, Germany; Natural History Museum of Denmark, Denmark

## Abstract

Cell-free DNA (cfDNA) in body tissues or fluids is extensively investigated in clinical medicine and other research fields. In this article we provide a direct quantitative real-time PCR (qPCR) as a sensitive tool for the measurement of cfDNA from plasma without previous DNA extraction, which is known to be accompanied by a reduction of DNA yield. The primer sets were designed to amplify a 90 and 222 bp multi-locus L1PA2 sequence. In the first module, cfDNA concentrations in unpurified plasma were compared to cfDNA concentrations in the eluate and the flow-through of the QIAamp DNA Blood Mini Kit and in the eluate of a phenol-chloroform isoamyl (PCI) based DNA extraction, to elucidate the DNA losses during extraction. The analyses revealed 2.79-fold higher cfDNA concentrations in unpurified plasma compared to the eluate of the QIAamp DNA Blood Mini Kit, while 36.7% of the total cfDNA were found in the flow-through. The PCI procedure only performed well on samples with high cfDNA concentrations, showing 87.4% of the concentrations measured in plasma. The DNA integrity strongly depended on the sample treatment. Further qualitative analyses indicated differing fractions of cfDNA fragment lengths in the eluate of both extraction methods. In the second module, cfDNA concentrations in the plasma of 74 coronary heart disease patients were compared to cfDNA concentrations of 74 healthy controls, using the direct L1PA2 qPCR for cfDNA quantification. The patient collective showed significantly higher cfDNA levels (mean (SD) 20.1 (23.8) ng/ml; range 5.1–183.0 ng/ml) compared to the healthy controls (9.7 (4.2) ng/ml; range 1.6–23.7 ng/ml). With our direct qPCR, we recommend a simple, economic and sensitive procedure for the quantification of cfDNA concentrations from plasma that might find broad applicability, if cfDNA became an established marker in the assessment of pathophysiological conditions.

## Introduction

The phenomenon of increased concentrations of circulating cell-free DNA (cfDNA) is considered a hallmark of various pathologic conditions like cancer, autoimmune diseases, stroke, sepsis, trauma and myocardial infarction (for review see [1], [2]). Intense exercise like half- and ultra-marathon running [3], [4], weight-lifting [5], [6] or an incremental treadmill-test until exhaustion [7], has also been shown to increase cfDNA concentrations in the plasma of healthy persons (for review see [8]). Until now, the entire mechanism of cfDNA increases has not been elucidated. In many clinical issues, it is assumed that the cfDNA fragments result from necrosis and apoptosis of blood and tissue cells [9]. Further possible mechanisms to explain more spontaneously occurring accumulations of cfDNA might be an active cfDNA release of extracellular or intracellular DNA [10]–[12], leukocyte oxidative burst [13] or extracellular trap formation [7].

It is hoped that quantitative and qualitative characteristics of cfDNA fragments in body tissues or fluids might be established as prognostic or diagnostic markers in medicine. The current procedures for quantitative cfDNA analysis imply DNA purification with subsequent absolute quantification of various DNA sequences by quantitative real-time PCR (qPCR). Unfortunately, the DNA extraction is accompanied by several constraints: i) DNA isolation methods require large amounts of blood samples [14]. ii) DNA isolation is time-consuming and increases the risk of contamination or inconsistency. Ingredients used for purification may contain PCR inhibitors minimizing the assay resolution or leading to false quantitative or qualitative outcomes [15]. iii) The DNA yield is reduced to an uncertain extent, depending on the isolation method of choice and the fragment size of the quested DNA [16]–[21]. This in turn might shift the DNA integrity values. The comparability of absolute cfDNA values from different studies is therefore limited.

One approach to overcome the mentioned aspects restricting cfDNA measurement is quantifying cfDNA directly in plasma or serum, like it has been recommended recently by Schwarzenbach et al. [2]. Umetani et al. [22]–[24] already published a method for cfDNA quantification without preceding DNA extraction. Before qPCR measurement, the plasma or serum samples were treated with a preparation buffer and proteinase K to remove proteins. The absolute quantification of cfDNA was implemented by the amplification of two lengths of ALU sequences. However, other groups used the ALU primers from this work, but not the direct qPCR procedure [25]–[27].

In this paper, we demonstrate a new, direct qPCR procedure for the sensitive quantification of cfDNA fragments from unpurified plasma in two modules. The reaction mixture of the direct qPCR contained a special polymerase, produced for the “amplification of difficult templates” (according to the product catalog 2011/12 from Bioline Ltd.). The primer sets were designed to amplify two lengths of a multi-locus L1PA2 consensus sequence. L1PA2 is a human Long Interspersed Element (LINE) of the class L1, that is well interspersed throughout the human genome [28]–[30].

In Module 1, cfDNA concentrations in unpurified plasma were compared to cfDNA concentrations in the eluate of a gel membrane and of a phenol-chloroform isoamyl (PCI) based DNA purification procedure using the L1PA2 approach, to elucidate the veritable DNA loss during extraction. For the comparison of the cfDNA concentrations determined by multi-locus L1PA2 amplification, the purified samples were also quantified for a single-locus repeat.

For the assessment of the applicability of the direct L1PA2 qPCR, further empirical data were presented in Module 2. cfDNA concentrations in the plasma of patients suffering from the coronary heart disease (CHD) were compared to cfDNA levels in healthy controls using the introduced direct L1PA2 qPCR procedure for cfDNA quantification. CHD comprises arteriosclerotic narrowing of the coronary vessels that supply blood and oxygen to the heart, leading to oxidative stress including inflammatory processes and ischemia-induced myocardial damage [31]. Acute events in patients with CHD occur either as angina pectoris, myocardial infarction (MI) or the acute coronary syndrome (ACS). In cases of MI and ACS, cfDNA concentrations have been shown to be significantly elevated compared to healthy persons, with cfDNA concentrations being either associated with the severity of the clinical condition or with the prognosis of the patients [32]–[34]. However, we are not aware of empirical data on cfDNA concentrations of patients with assessed CHD that do not suffer from acute medical conditions at the time point of sampling. We hypothesized that if CHD patients exhibited elevated cfDNA concentrations, cfDNA might serve as a marker for regular monitoring, to give opportunely evidence when the myocardial oxygen supply becomes critical.

## Methods

### Ethical approval

All experimental procedures were approved by the Human Ethics Committee Baden Württemberg for Module 1 and by the Human Ethics Committee of Rhineland-Palatine for Module 2, and conformed to the standards of the *Declaration of Helsinki of the World Medical Association*. The subjects gave written consent to participate in the study.

### Subjects, blood sampling and processing

#### Module 1. Establishment of the direct L1PA2 qPCR

Before and after a 10 km relay race, 2.7 µl of EDTA-coagulated blood were collected from 10 recreational runners (6 male, 4 female, mean (SD) age 36.0 (14.3) years, height 178.4 (10.3) cm, weight 74.1 (11.3) kg). Plasma was obtained by centrifugation of the whole blood samples at 4°C and 1,600*g for 10 minutes. In a second step the supernatants were microcentrifuged at 4°C and 16,000*g for 5 min to remove cell debris from the plasma [7], [35]. The plasma was diluted in sterile water in a 1∶40 ratio for direct qPCR measurement. The diluted samples and the remaining plasma volumes were stored at −20°C until measurement or further treatment.

For the validation of the new direct qPCR, 100 µl of plasma were isolated with the QIAamp DNA Blood Mini Kit (Qiagen GmbH, Hilden, Germany) according to producers' instructions and eluted in a final volume of 100 µl TE buffer. The purified DNA was measured by a qPCR targeting an 88 bp fragment of the single-locus gene myostatin (*MSTN*; Table 1; the MSTN qPCR is introduced in the supplemental section “Methods S1” and described in detail in [7]) and with the L1PA2 qPCR in a 1∶40 dilution in TE buffer. To investigate the loss of cfDNA during the gel-membrane based isolation procedure, the flow-through of all washing steps was collected in a tube for each sample. The flow-through was diluted 1∶40 in H_2_O and measured according to the introduced L1PA2 qPCR protocol.

**Table 1:** qPCR and PCR conditions.

	L1PA2 qPCR	MSTN qPCR	PCR for standard generation
Kit/Polymerase	Velocity Polymerase (Bioline, Luckenwalde, Germany)	QuantiFast SYBR Green PCR Kit (Qiagen GmbH, Hilden, Germany)	HotStar HiFidelity PCR Kit (Qiagen GmbH, Hilden, Germany)
Primer sequence forward	5′-TGCCGCAATAAACA TACGTG-3′ (90 bp and 222 bp amplicon)	5′-TTGGCTCAAACAACCT GAATCC-3′	5′-GGAGCAGACAAGCCC GTCAGG-3′
Primer sequence reverse	90 bp amplicon: 5′-GACCCAGCCATCCC ATTAC-3′; 222 bp amplicon: 5′-AACAACAGGTGCTG GAGAGG-3′	5′-TCCTGGGAAGGTTACA GCAAG-3′	5′-CAGGCTTTACACTTTA TGCTTCC GGC-3′
Final primer concentrations (each primer; µM)	0.34	0.30	1.00
Annealing (°C)	64	59	60
Cycle numbers	35	40	40

To overcome the losses during gel-membrane based DNA isolation, 100 µl of plasma were purified using phenol-chloroform isoamyl (PCI) and finally diluted in 20 µl TE buffer (the protocol for DNA purification with PCI is described in “Methods S1”).

#### Module 2. Implementation of the direct qPCR for the comparison of cfDNA concentrations in diseased subjects and controls

EDTA-coagulated blood was collected from the fingertips of 74 subjects suffering from CHD (60 male, 14 female, age 61.5 (8.6) years, height 173.5 (7.6) cm, weight 90.9 (14.3) kg) and 74 healthy controls (53 male, 21 female, age 37.5 (14.7) years, height 177.7 (8.8) cm, weight 79.1 (13.6) kg). The CHD patients were recruited from the University Medical Center Mainz and either suffered from recurring or chronic angina pectoris or had already suffered myocardial infarction. The control group consisted of healthy volunteers. Plasma was obtained as described above by slow (1,600*g, 10 min, 4°C) and subsequent high-speed centrifugation (16,000*g, 5 min, 4°C) and diluted in a 1∶40 ratio in H_2_O. The cfDNA concentrations were directly quantified from the unpurified plasma using the L1PA2 qPCR procedure.

### Quantitative real-time PCR reaction and conditions

cfDNA concentrations in plasma were quantified by a direct real-time PCR method (Table 1). For the determination of test and software variations all templates were measured in triplicates. 6.4 µl of diluted plasma were added as template to 41.6 µl master mix containing 1 U/µl Tego Buffer (Bioline, Luckenwalde, Germany), 0.05 U/µl Velocity Polymerase (Bioline, Luckenwalde, Germany), 0.17× SYBR Green (Sigma-Aldrich Co., Taufkirchen, Germany), 0.001 µM FITC (Sigma-Aldrich Co., Taufkirchen, Germany), 0.6 mM MgCl_2_ and 0.34 µM each primer. The total volume of reaction mixture per template was 48 µl adequate to three measurements of 15 µl plus pipetting loss. The final plasma volume per well was 0.05 µl. All reactions comprised a triplicate of non-template controls (NTC).

Reactions were carried out in 96-well PCR plates (0.2 ml tube plate, white, Peqlab, Erlangen, Germany) using the iCycler MyIQ Detection System (Biorad, Munich, Germany) for qPCR measurement. Amplification consisted of an initial denaturation for 2 min at 98°C, followed by 35 cycles of melting at 94°C for 10 s, annealing at 64°C for 40 s and extension at 75°C for 10 s. Subsequent qPCR measurements were calibrated to an interplate-calibration template that had been measured several times on plates containing a standard dilution series.

### Primer design

L1PA2 sequence information for primer design, matches with the human genome, and custom DNA design for standard curves were requested from the UCSC genome browser (http://genome.ucsc.edu). The primer sets and a L1PA2 custom DNA sequence were designed with Primer3 (http://frodo.wi.mit.edu/primer3/; [36]). For the determination of the DNA integrity as a qualitative measure, L1PA2 primer sets were established for a 90 bp and a 222 bp amplicon, showing 3345 and 3134 matches in the human genome, respectively. The 90 bp primers were implemented for the amplification of the entire cfDNA fragments in the samples, while the 222 bp sequence should represent only the fraction of larger cfDNA fragments [37]. The primer sequences for the L1PA2 90 bp fragment were as forward, 5′-TGCCGCAATAAACATACGTG-3′ and reverse, 5′-GACCCAGCCATCCCATTAC-3′. For the 222 bp L1PA2 amplicon primers were as forward 5′-TGCCGCAATAAACATACGTG-3′, which is the same as for the 90 bp amplicon, and as reverse, 5′-AACAACAGGTGCTGGAGAGG-3′ (Table 1). The DNA integrity was calculated as the quotient of L1PA2 222 bp qPCR results and 90 bp qPCR results.

### L1PA2 custom DNA standard sequence

The 401 bp L1PA2 custom DNA sequence was synthesized in a pEx-A plasmid by Eurofins MWG Operon (Ebersberg, Germany). The plasmids were inserted in a PCR using the HotStar HiFidelity PCR Kit (Qiagen GmbH, Hilden, Germany) according to the manufacturer's instructions (further information in Table 1), for the amplification of a 629 bp sequence including the L1PA2 sequence between two pEx primer sequences. The PCR product was applied on a 1.5% agarose gel to separate the plasmids from the 629 bp L1PA2 amplicon. An approximately 600 bp band was cut and purified with the QIAquick Gel Extraction Kit (Qiagen GmbH, Hilden, Germany). In a second PCR the L1PA2 amplicon was manifolded again. The final L1PA2 sequence in the PCR product was purified using the Fermentas GeneJET PCR Purification Kit (Thermo Fisher Scientific Inc., Dreieich, Germany) and diluted in 60 µl TE buffer. The concentration of L1PA2 fragments in the stock solution was measured using a fluorospectrometric technique (NanoDrop 3300, Thermo Fisher Scientific Inc., Dreieich, Germany). The program Finnzymes (www.finnzymes.com/java_applets/copy_number_calculation.html) was used to calculate the initial copy number of the calibrator dilution in the standard series.

### Standard curves and limit of quantification

For an absolute quantification of cfDNA fragments, a standard curve of serially diluted concentrations of the L1PA2 custom DNA fragments was established. The final standard series contained serial dilutions of defined L1PA2 copy numbers in TE buffer, ranging from 10^6^ to 75 copies/2 µl, with one copy being a double-stranded DNA fragment in a haploid cell. 2 µl of the standard dilutions were applied per well for qPCR standard curve establishment. In each dilution the measured threshold cycle value was plotted against the logarithm of the calibrator copy number [38]. The L1PA2 standard dilution series was first implemented purely in the qPCR for the confirmation of the qPCR efficiency that was determined as proposed by Mygind et al. [39] for the validation of the new approach. Since the unpurified plasma templates in the direct measurement contained proteins, minerals and EDTA that might inhibit or tarnish the reaction, murine plasma (0.05 µl per well) was implemented in a second standard curve amplification to meet the conditions of template measurement.

For the detection of the lowest limit of quantification (LOQ) dilutions from 1400 to 75 copies/2 µl spiked with murine plasma were measured as septets. The LOQ was defined as the lowest copy number that revealed not more than 20% standard deviation in a septet of a standard dilution [7].

In the next step, a dilution series of a human plasma sample was implemented to further validate the PCR efficiency in unpurified sample material. The initial template (plasma diluted in H_2_O in a ratio of 1∶40 and quantified by qPCR measurement) contained 271 ng of the 90 bp cfDNA fragments per ml plasma and was serially diluted in 8 steps in a ratio of 1∶2. The H_2_O diluents contained murine plasma in a ratio of 1∶40, so that each final dilution contained the same total amount of plasma, while the portion of human plasma was halved in each step. All templates were amplified using the 90 bp and the 222 bp L1PA2 primers.

### Qualitative analysis of cfDNA

The eluate of the cfDNA extractions with the QIAamp DNA Blood Mini Kit and the PCI procedure in Module 1 were investigated for DNA fragment sizes using the Fragment Analyzer™ (Advanced Analytical Technologies GmbH, Heidelberg, Germany). The dsDNA Reagent Kit DNF-910 (Advanced Analytical Technologies GmbH, Heidelberg, Germany) was used for qualitative analysis of cfDNA fragments ranging from 35 to 1500 bp. Normalization was performed using defined markers for 35 and 1500 bp fragments, respectively. Due to the low sample concentration, sample injection was performed at 5.0 kV for 180 seconds. The separation during electrophoresis was executed at 6.0 kV for 40 minutes.

### Statistical analysis

The qPCR data were captured with the MyIQ5 Optical System Software, Version 2.4 (Biorad, Munich, Germany). Microsoft® Excel 2007 (Microsoft Corp., Redmond, WA, USA) was used for data capturing and descriptive figures. Statistical analyses were performed with JMP 8 (SAS Institute Inc., Cary, NC, USA). All data were presented as mean and standard deviation. Since the overall data were not normally distributed, a Spearman's rho test was calculated for nonparametric correlations. For the data analyzed in Module 1 the separation by pre exercise and post exercise resulted in a normal distribution and allowed the calculation of a linear regression analyses model for the comparison of absolute cfDNA concentrations measured for the MSTN and L1PA2 amplicon or the plasma and eluate. Changes of cfDNA concentrations from pre exercise to post exercise were calculated as mean fold-differences. The fold-differences and the DNA integrity values met a normal distribution and were analyzed for significant correlations using the Pearson's correlation and for mean differences using the Student's t test for paired samples. For Module 2 the cfDNA concentrations in the plasma of CHD patients and healthy controls were compared with the Student's t-test for independent samples. To meet the criteria of normal distribution, the cfDNA concentrations were converted to logarithmic data.

## Results

### Module 1. Establishment of the direct L1PA2 qPCR

#### Linearity and Sensitivity of the L1PA2 qPCR

**Figure 1:**
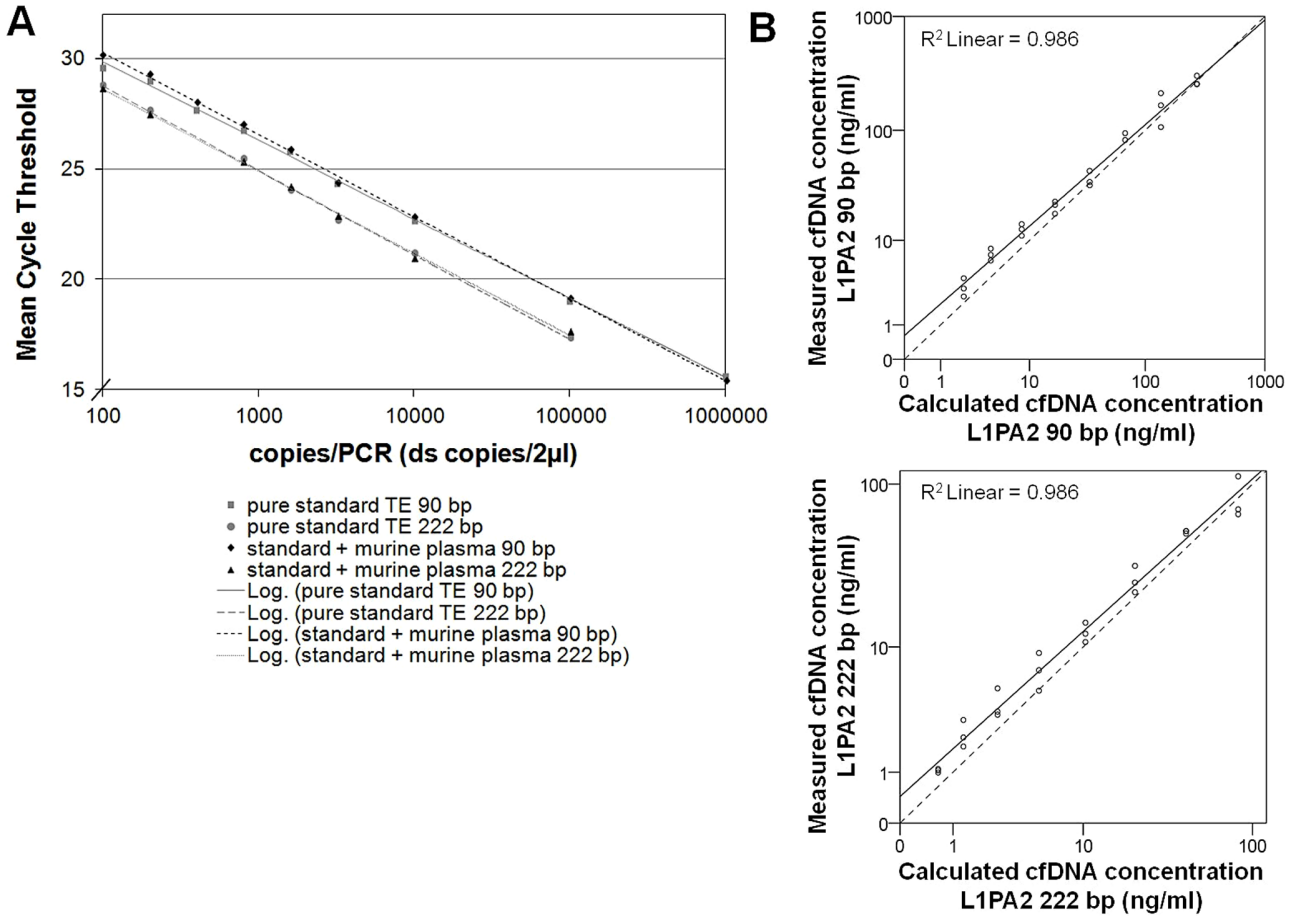
Linearity of the L1PA2 qPCR. A. Linearity in the standard curve of L1PA2 90^6^ to 100 copies per reaction in TE and in mixtures spiked with murine plasma, to meet the characteristics of the direct measurement of cfDNA in human plasma. The qPCR efficiency was not affected by the ingredients of murine plasma. B. Dilution series of a human plasma cfDNA sample: the correlation of calculated and measured concentrations (upper: L1PA2 90 bp amplicon; lower: L1PA2 222 bp amplicon) shows a slight drift as a result of the qPCR efficiency of 93.97% that was applied for the calculations of absolute concentrations (solid line = regression line; dashed line = angle bisector).

For the L1PA2 90 bp amplicon, the standard curve showed linearity ranging from 106 to 100 copies per reaction with a PCR efficiency of 95.19% for the pure standard mixture and 93.97% for the standards spiked with murine plasma. Linear regression analysis of mean cycle threshold values per triplicate against log concentrations in the dilution yielded *R*>0.99. The standard curve of the L1PA2 222 bp amplicon revealed a PCR efficiency of 91.37% and 92.99% and a regression coefficient of *R*>0.99 (Fig. 1 A). Both standard curves spiked with murine plasma showed equivalent axis and slope compared to the pure standard curves, indicating that the murine plasma did not affect the qPCR efficiency. For both L1PA2 amplicon lengths the LOQs were determined at 100 copies per reaction.

The dilution series of the human plasma sample showed linearity over the whole range. PCR efficiency for the mean cycle threshold values was calculated 100.3% for the L1PA2 90 bp fragments (R>0.99) and 99.9% for the L1PA2 222 bp fragments (R>0.99). Figure 1 B shows the scatter plot of the measured concentrations and the estimated concentrations that were calculated based on the measured initial concentration. The slide drift of the regression line was caused by the qPCR efficiency of the standard curve that was used to convert the measured cycle threshold values into absolute cfDNA concentrations in ng/ml.

#### Comparison of single and multi locus qPCR

**Figure 2:**
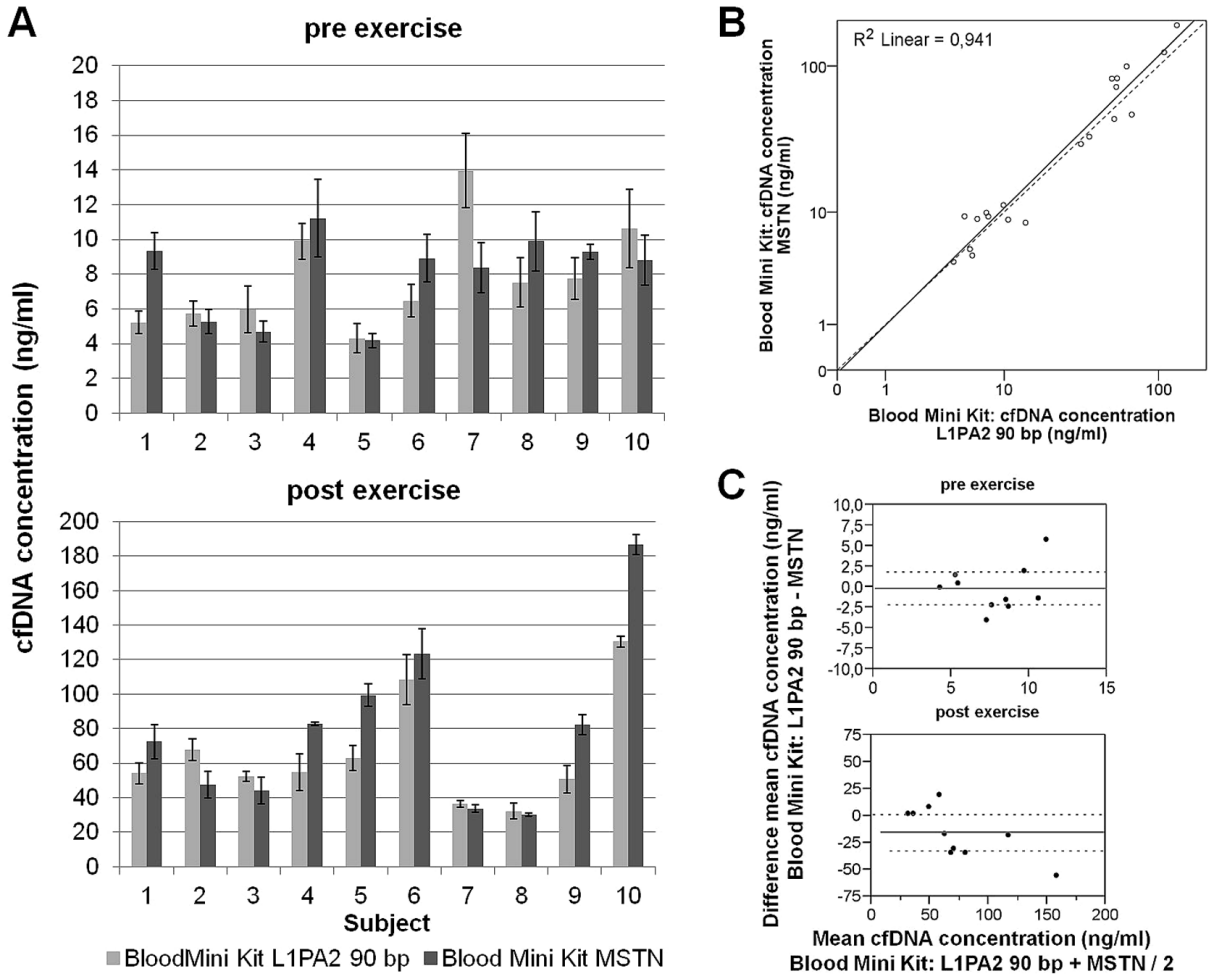
Comparison of the qPCR results from the multilocus L1PA2 and the single-locus MSTN sequence amplification. A. Absolute cfDNA concentrations measured with the L1PA2 and the MSTN qPCR in the eluate of DNA purification with the QIAamp DNA Blood Mini Kit (mean concentrations and standard deviations), showing a good concordance between amplifications of single and multi-locus repeats. B. Correlation of cfDNA concentrations (mean triplicate values). C. Paired differences for mean concentrations measured for the L1PA2 and the MSTN repeats.

Amplification of the abundant L1PA2 90 bp sequence and the MSTN single gene locus revealed comparable cfDNA concentrations in the eluate of isolated DNA with the QIAamp DNA Blood Mini Kit (Fig. 2 A). An overall analysis of cfDNA concentrations pre and post exercise revealed a Spearman's rho correlation coefficient of r = 0.899 (p≤0.01; Fig. 2 B, C). Separate linear regression analysis of pre and post values only yielded a significant correlation between the L1PA2 and MSTN qPCR for post exercise concentrations (r = 0.910; p = 0.003).

Mean (SD) cfDNA concentrations before exercise were 7.75 (2.96) ng/ml and 7.91 (2.40) ng/ml determined by L1PA2 and MSTN qPCR, respectively. The mean difference between multi-locus and single-locus measurements was −0.16. For cfDNA concentrations post exercise the L1PA2 qPCR measured 65.0 (31.05) ng/ml. The MSTN qPCR revealed a mean concentration of 80.37 (47.96), resulting in a mean difference of −15.37. For both time points the student's t test showed no significant difference between both qPCR procedures (pre, t(9) = −0.178; p = 0.863; post, t(9) = −2.065; p = 0.069).

The fold-increases of cfDNA from pre exercise to post exercise measured in the eluate of the QIAamp DNA Blood Mini Kit with both qPCRs correlated significantly (r = 0.762; p = 0.01; L1PA2, 9.35 (4.62)-fold; MSTN, 10.86 (6.89)-fold). There was no significant difference between the fold-differences (t(9) = −1.062; p = 0.316).

#### Direct cfDNA measurement in plasma

**Figure 3:**
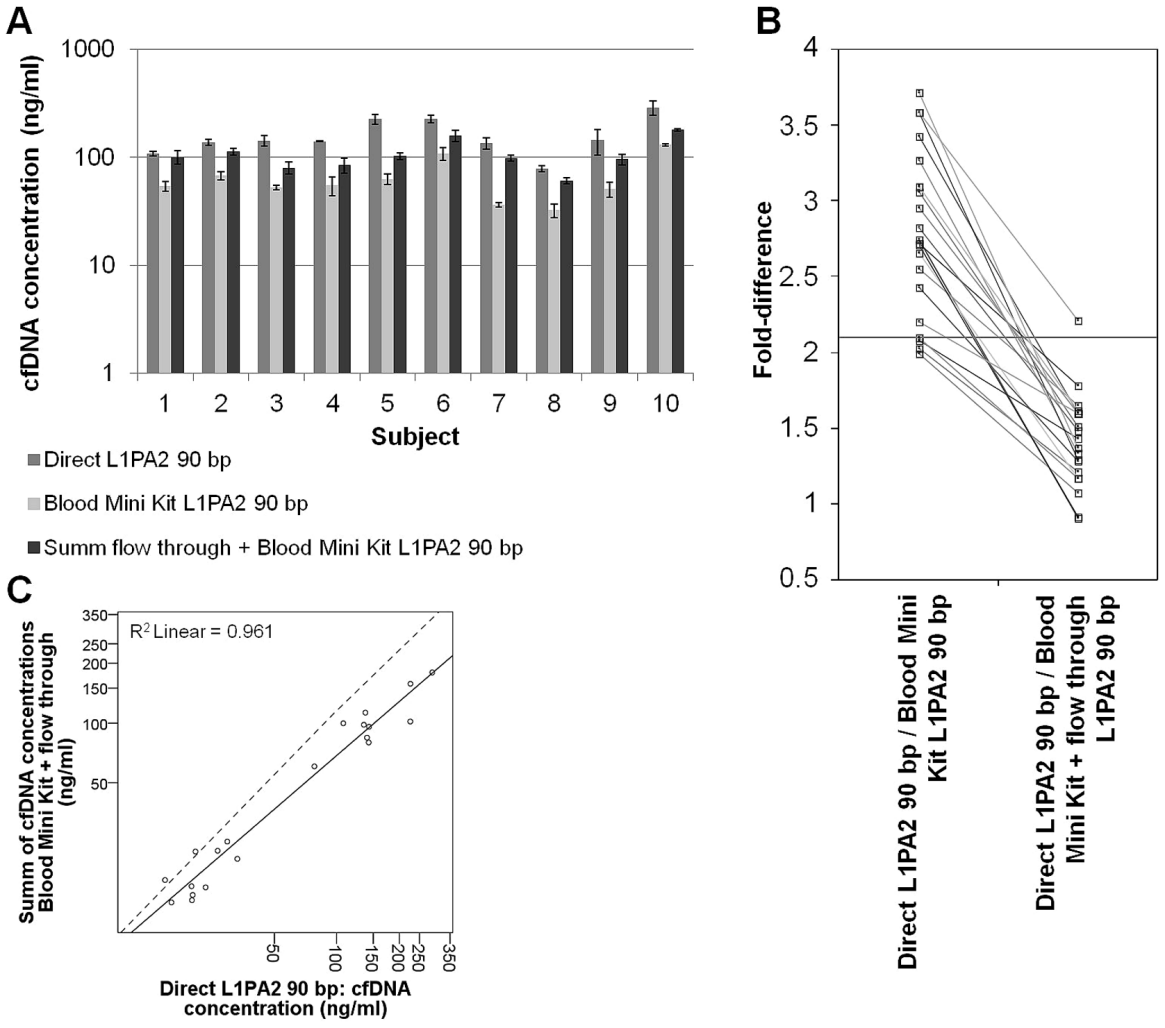
Comparison of cfDNA concentrations measured in unpurified and purified plasma. A. Absolute cfDNA concentrations post exercise measured with the L1PA2 qPCR in plasma and in the eluate and flow-through of the QIAamp DNA Blood Mini Kit. The concentrations determined in the flow-through were summed up with the concentrations in the eluate (sum_FBM_). B. Fold-difference between the absolute concentrations measured directly in plasma compared to the eluate of the QIAamp DNA Blood Mini Kit and to the sum_FBM_. C. Scatter plot showing the Spearman's correlation of cfDNA concentrations in plasma and the sum_FBM_ (solid line = regression line; dashed line = angle bisector).

cfDNA concentrations measured directly in plasma were compared to cfDNA concentrations measured in the eluate of the QIAamp DNA Blood Mini Kit. Both qPCR measurements were performed with the Velocity Polymerase master mix and L1PA2 primers amplifying the 90 bp amplicon. For pre exercise values, the qPCR revealed 2.96 (0.43)-fold higher cfDNA concentrations in plasma (22.09 (5.91) ng/ml) compared to the eluate (7.75 (2.96) ng/ml). In post exercise templates, the direct measurement resulted in 2.62 (0.62)-fold higher cfDNA concentrations compared to the eluate (162.40 (63.65) ng/ml in plasma; 65.0 (31.05) ng/ml in the eluate; Fig. 3 A, B). The differences between the templates were significant for both time points (pre, t(9) = 12.901; post, t(9) = 7.862; p≤0.0001). Separate linear regression analyses of pre and post exercise cfDNA concentrations yielded a significant correlation between concentrations measured directly in plasma and in the eluate (pre, r = 0.897; p = 0.0004; post, r = 0.881; p≤0.0008). A Spearman's rho correlation for pre and post exercise samples revealed r = 0.959 (p≤0.01). Comparison of the fold-increases from pre exercise to post exercise determined in plasma (7.65 (3.26)-fold) and the eluate (9.35 (4.62)-fold) revealed a significant difference between the templates (t(9) = −2.280; p = 0.049).

The flow-through of the whole isolation procedure was collected for each sample and measured in the L1PA2 qPCR, to investigate the loss of cfDNA during the gel-membrane based DNA extraction. The measurement showed that a substantial amount of cfDNA fragments is present in the waste material of the silica based isolation. The sum of cfDNA concentrations in the flow-through and in the eluate of the QIAamp DNA Blood Mini Kit (sum_FBM_) revealed nearly as high values (pre, 17.17 (4.81); post, 107.14 (35.83) ng/ml; Fig. 3 A) as measured directly in plasma samples. The cfDNA concentrations in plasma remained 1.42 (0.31)-fold higher (Fig. 3 B, C). Since the flow-through templates were highly diluted, most of the lower concentrated pre exercise templates yielded threshold cycle values outside the LOQ. Therefore, further statistical analysis was performed only for post exercise values. The sum_FBM_ and plasma cfDNA concentrations correlated significantly (r = 0.860; p = 0.0012). The linear regression analysis for post exercise cfDNA concentrations still gave a significant difference between the plasma and the sum_FBM_ values (t(9) = 4.65; p = 0.01).

cfDNA concentrations measured directly in plasma were compared to cfDNA concentrations in the eluate of a PCI based DNA extraction. The analysis showed a different ratio between the templates in low or high cfDNA concentrations. For pre exercise values, the qPCR revealed 3.36 (2.74)-fold higher cfDNA concentrations in plasma (22.09 (5.91) ng/ml) compared to the eluate (8.79 (4.06) ng/ml), giving no significant correlation. The post exercise values of direct und PCI measurement correlated well (r = 0.891; p = 0.0005), with plasma cfDNA concentrations being only 1.31 (0.53)-fold higher compared to the eluate (162.40 (63.65) ng/ml in plasma; 148.45 (90.77) ng/ml in the eluate).

#### DNA integrity before and after exercise

**Figure 4:**
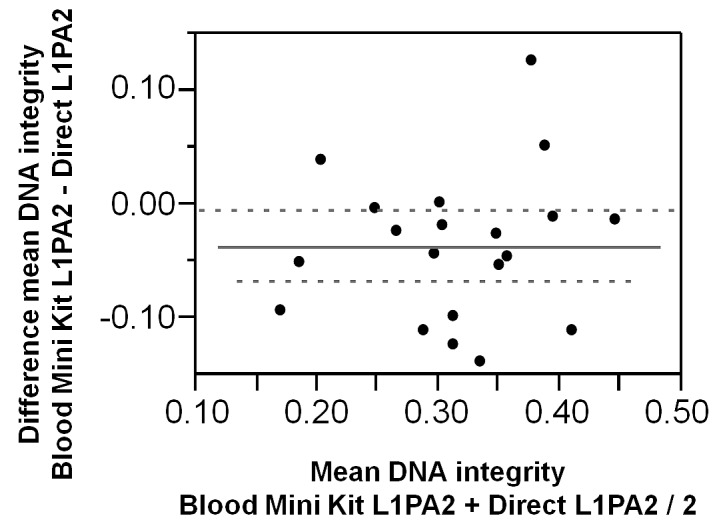
DNA integrity in unpurified and purified plasma samples. Paired differences of DNA integrity values measured directly in plasma and in the eluate of the QIAamp DNA Blood Mini Kit.

The DNA integrity decreased in the plasma and in the eluate of both DNA extraction procedures from pre exercise to post exercise levels (Table 2). The integrity values determined in plasma and in the eluate of the QIAamp DNA Blood Mini Kit were similar (pre, r = 0.26; p = 0.001; post, r = 0.85; p = 0.002) and did not differ significantly from each other (pre, t(9) = 1.934; 0.085; post, t(9) = 1.642; p = 0.135). Analysis of the paired differences of DNA integrities in plasma and in the eluate of the QIAamp DNA Blood Mini Kit revealed a slight but significant difference for both points in time pre and post exercise (Fig. 4). The eluate of the PCI extraction yielded considerably lower DNA integrity values.

**Table 2:** DNA integrity values pre and post exercise measured directly in plasma and in the eluate of the QIAamp DNA Blood Mini Kit and the PCI DNA extraction.

	Pre (mean (SD))	Post (mean (SD))
Plasma (direct)	0.376 (0.054)	0.288 (0.072)
Eluate QIAamp DNA Blood Mini Kit	0.326 (0.076)	0.264 (0.085)
Eluate PCI	0.185 (0.050)	0.175 (0.057)

#### Fragment lengths of cfDNA

**Figure 5:**
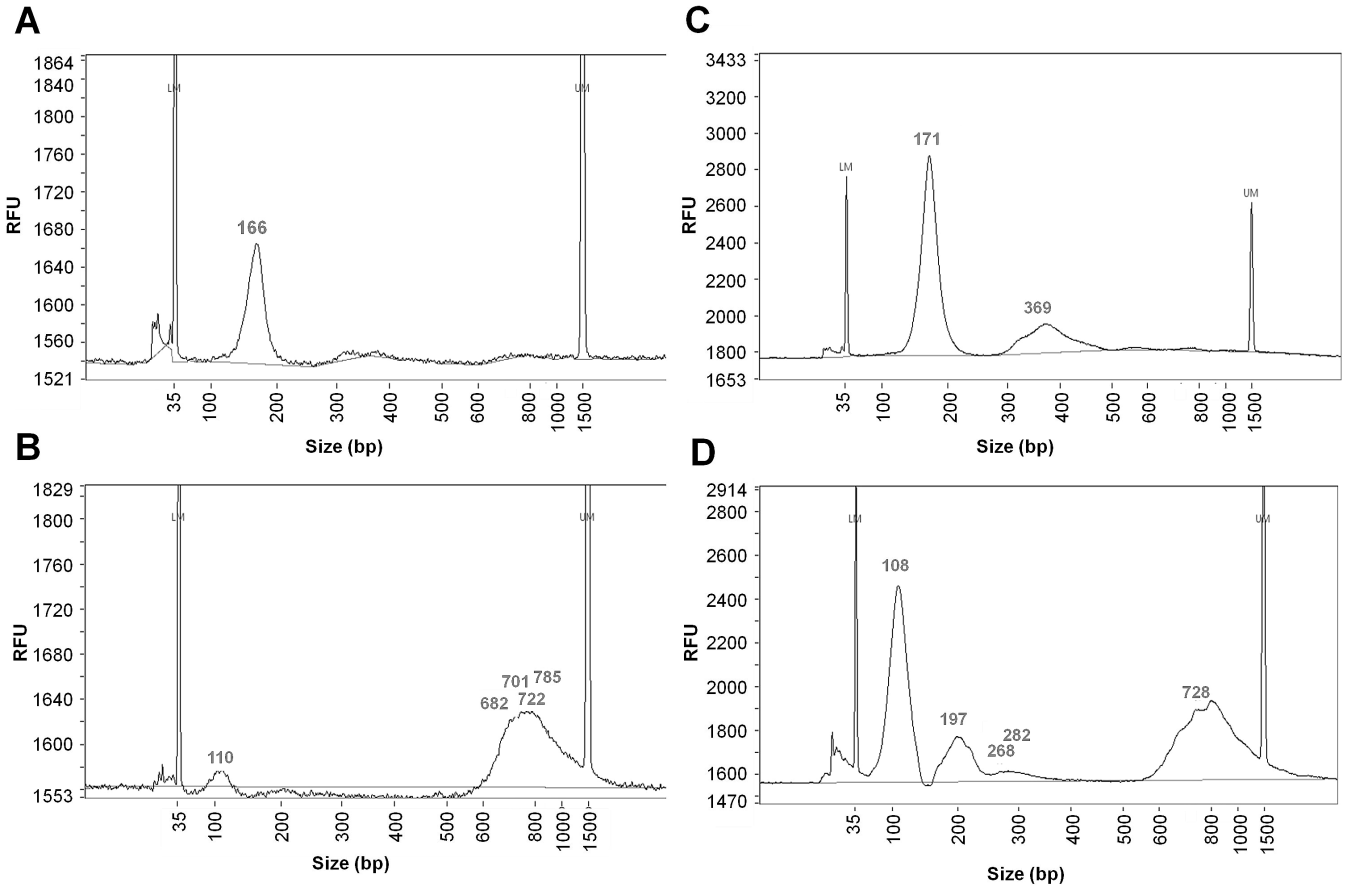
Data from the Fragment Analyzer™ measurement shown by an example of one subject. A. Sampling pre exercise and DNA extraction with the QIAamp DNA Blood Mini Kit; B. Sampling pre exercise and DNA extraction with PCI procedure; C. Sampling post exercise and DNA extraction with the QIAamp DNA Blood Mini Kit; D. Sampling pre exercise and DNA extraction with PCI procedure (LM = lower marker (35 bp); UM = upper marker (1500 bp)).

**Figure 6:**
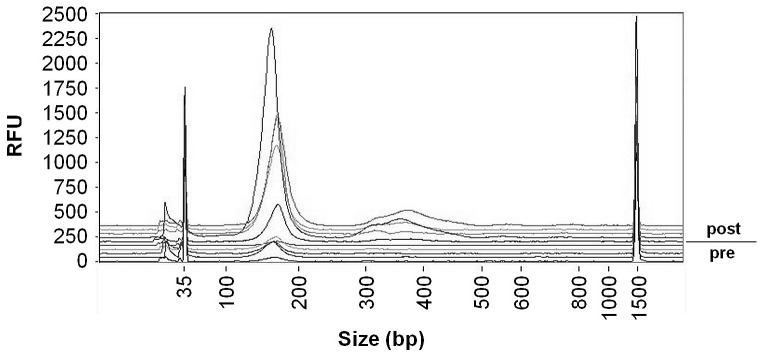
Overlay graphic of Fragment Analyzer™ data from 5 subjects pre and post exercise. The samples presented here were purified using the QIAamp DNA Blood Mini Kit. At both time points, pre and post exercise, all samples showed a peak at approximately 170 bp. Post exercise, the concentrations of the this fragment length increased and a second peak was evident at on average 360 bp of length.

For both points in time, the eluate of the QIAamp DNA Blood Mini Kit and the PCI extraction were analyzed for fragment size distributions. Figure 5 displays the results of the Fragment Analyzer™ measurement from one subject for both extraction methods (A–D) and Figure 6 presents an overlay graphic from 5 subjects for the QIAamp DNA Blood Mini Kit, pre and post exercise, respectively. All measurements of cfDNA fragments were characterized by broad peaks corresponding to a light smear.

In the eluate of the QIAamp DNA Blood Mini Kit all subjects showed a peak at an average of 167 (3.4) bp pre exercise. In four templates, we also found larger cfDNA fragments with two templates containing an average of 352 (4.2) bp and three templates peaking at an average of 627 (21.7) bp. Post exercise, the concentrations of the short fragment lengths of about 170 bp increased in all subjects. Furthermore, all post exercise templates of the silica-membrane based DNA extraction indicated a peak at an average of 360 (18.0) bp, with four templates showing another peak at 656 (94.0) bp.

The eluate of the PCI extraction revealed a differing pattern of fragment lengths compared to the eluate of the QIAamp DNA Blood Mini Kit. All templates drawn before exercise exhibited a peak for short cfDNA fragments at an average of 107 (2.4) bp and for long fragments at an average of 710 (73.2) bp. Post exercise, the concentrations of the short fragment lengths of about 110 bp increased and a peak at 196 (3.2) bp occurred, like it had been shown for the eluate of the QIAamp DNA Blood Mini Kit. In three samples, another peak appeared for a length of 278 (8.3) bp. Table 3 presents the peak lengths of cfDNA fragments in all templates, which were categorized into four groups of fragment sizes (<160 bp; 160–210 bp; 210–500 bp; >500 bp). For each category, the table displays the mean value and the distribution of cfDNA fragment lengths as well as the number of templates (N) that presented the respective peaks.

**Table 3:** Mean (SD) and distributions of cfDNA fragment lengths measured with the Fragment Analyzer™ in the eluate of the QIAamp DNA Blood Mini Kit and the PCI DNA extraction.

Fragment length	Dimension	Eluate QIAamp DNA Blood Mini Kit		Eluate PCI	
		pre exercise	post exercise	pre exercise	post exercise
<160	Mean (SD)		98 (0)	108 (2.4)	108 (1.1)
	Distribution		98	104–111	107–110
	N		1	10	10
160–210	Mean (SD)	167 (3.4)	168 (4.1)		196 (3.2)
	Distribution	160–172	160–172		194–202
	N	10	10		10
211–500	Mean (SD)	352 (4.2)	360 (18.0)		278 (8.3)
	Distribution	349–355	332–378		268–287
	N	2	10		3
>500	Mean (SD)	647 (21.7)	656 (94.4)	710 (73.2)	712 (62.5)
	Distribution	627–670	552–749	623–950	651–781
	N	3	4	10	10

### Module 2. Implementation of the direct qPCR for the comparison of cfDNA concentrations in diseased subjects and controls

**Figure 7:**
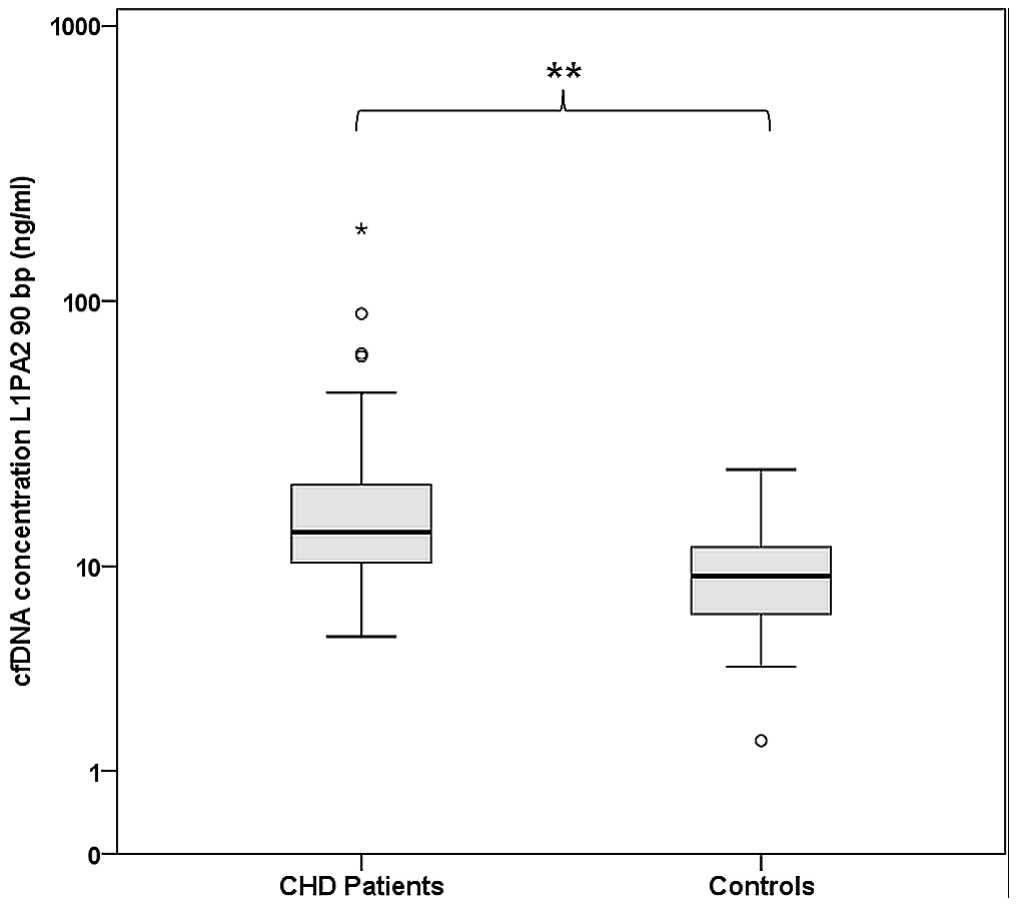
Comparison of cfDNA concentrations measured in 74 CHD patients and 74 healthy controls. cfDNA concentrations were significantly higher in the patient collective. The data were presented as absolute cfDNA concentrations (ng/ml), while logarithmic data were inserted for the comparison of means.

For the presentation of empirical data, plasma cfDNA concentrations were analyzed from 74 CHD patients and 74 healthy controls using the introduced direct qPCR procedure. The CHD patients showed a mean (SD) cfDNA baseline level of 20.1 (23.8) ng/ml in a range of 5.1 to 183.0 ng/ml. Compared to the collective of patients, the healthy controls exhibited lower cfDNA values with a mean (SD) of 9.7 (4.2) ng/ml and a smaller range between a minimum of 1.6 ng/ml and a maximum of 23.7 ng/ml (Table 4; Fig. 7). The Student's t-test for independent samples revealed a highly significant difference between the logarithmic cfDNA values of both collectives (t(132.0) = 5.94; 95% CI, 4.9–16.0; p<0.001; Fig. 7), with mean cfDNA concentrations of the CHD patients being 2-fold higher compared to the controls.

**Table 4:** cfDNA concentrations measured directly in the unpurified plasma of CHD patients and healthy controls presented as mean (SD) levels, minimum and maximum values and percentiles.

Collective	N	Mean (SD) cfDNA (ng/ml)	Minimum cfDNA (ng/ml)	Maximum cfDNA (ng/ml)	Percentile 25 (ng/ml)	Percentile 50 (ng/ml)	Percentile 75 (ng/ml)
CHD	74	20.1 (23.8)	5.1	183.0	10.3	13.7	20.8
Controls	74	9.7 (4.2)	1.6	23.7	6.4	9.2	11.9

## Discussion

DNA purification methods have been proven to be time consuming and susceptible for errors or DNA losses [15], [18], [21]. In this article we provide a direct qPCR procedure as a sensitive tool for the quantification of cfDNA from plasma without previous DNA extraction that showed evident quantitative differences compared to a silica-based and a PCI procedure. The direct qPCR procedure has already performed well with plasma, serum or clear medium from *in vitro* cell cultures as templates (data from serum and medium experiments not shown).

Another approach for a direct qPCR procedure has already been published [22]–[24]. However, until today only the ALU primers have been adopted by other research groups from this work [25]–[27]. The disadvantage of this method might have been inherent in the ‘pre-procedure’ that comprised the addition of a preparation buffer and proteinase K, centrifugation and elution of the pellet and thus resembled a simplified variant of the PCI method. Our new procedure implies only one step before the template is ready to be inserted in the qPCR, which is a dilution of the plasma in H_2_O in a 1∶40 ratio.

The direct qPCR is based on the amplification of two lengths of an abundant L1PA2 repeat which is a subfamily of the LINEs. L1 elements constitute almost 17% of the human genome [40]. Since L1PA2 sequences are distributed over all chromosomes, the amplification of L1PA2 repeats might increase the sensitivity of cfDNA measurements [2]. Herrera et al. [16] stated that the amplification of multiple genomic loci might be advantageous in studies investigating solid tumors, because the loss or gain of a single-locus impacted the absolute cfDNA concentration less. However, Beck et al. [30] found L1 elements in a 20% higher proportion in fractions of genomic and cfDNA than expected from *in silico* analysis, but an underrepresentation of the L1 proportion (80%) in the fraction of cfDNA compared to genomic DNA. Furthermore, many of the L1 elements have been shown to be polymorphic in humans [29], unequally represented in the cfDNA of different individuals [41] and hypomethylated in cancer patients [42]. Our comparison of cfDNA concentrations quantified by the single-locus repeat of the MSTN gene and by L1PA2 repeats did not yield any significant differences. The higher mean differences for cfDNA concentrations post exercise might be partially caused by an individual underrepresentation of the L1PA2 repeats. Another reason might be the effect of the different qPCR efficiencies calculated for the single- and multi-locus primers to convert the cycle threshold values into absolute cfDNA concentrations, respectively.

The direct amplification of cfDNA in untreated plasma measured on average 2.79-fold higher cfDNA concentrations compared to the eluate of the QIAamp DNA Blood Mini Kit. These results were well in line with Herrera et al. [16] who found a mean recovery rate of 44.4% for this kit. It is assumed that approximately 5–10% of DNA fragments, especially smaller fragments <150 bp, stick to the silica membrane during elution [43], [44]. The quantification of cfDNA fragments in the flow-through of the QIAamp DNA Blood Mini Kit revealed that on average 36.7% of the total cfDNA were disposed with the high choatropic salt buffers. The cfDNA fragments might already get washed through the silica membrane during the first centrifugation step. Before the first centrifugation, a protease e.g. proteinase K is added to the plasma in order to cut the plasma proteins and to dissolve the bindings between the positive surface charges of the proteins and the negatively charged diester bonds of the DNA fragments, resulting in polypeptide and protein-unbound cfDNA fragments. After addition of a chaotropic salt buffer and ethanol the fluids are thoroughly mixed, transferred into a silica membrane tube and subsequently centrifuged. In the presence of the high salt concentrations, the DNA fragments are thought to completely bind to the positively charged silica membrane [45], [46]. It might be possible that, during the whole process, some polypeptide fragments remain or return positively charged, leading to polypeptide-DNA-complexes that do not stick to the silica gel but flow through the membrane instead.

The qualitative analysis in the eluate of the QIAamp DNA Blood Mini Kit indicated mean cfDNA fragment lengths of approximately 170 bp for baseline conditions. These results were in accordance with others [30], [47], [48] and correspond to the DNA strand length wrapped around a nucleosome plus linker DNA. Post exercise, the templates showed a second peak at around 350 bp. Heitzer et al. [48] found this dinucleosomal cfDNA fragment length in tumor patients, suggesting that this phenomenon might be a consequence of a saturation of DNA degradation mechanisms in the presence of high cfDNA concentrations. However, based on our results, we cannot support their assumption of the larger 350 bp fragments as being tumor specific.

For samples with high cfDNA concentrations, the PCI procedure revealed on average 87.4% of the concentrations measured in plasma. Unfortunately, the procedure did not perform well for low cfDNA concentrations in the plasma. Furthermore, the results provided by Fragment Analyzer™ measurements and DNA integrity analysis gave occasion to doubt the validity of the PCI procedure (see Fig. 5; Table 2).

On the other hand all fragment lengths detected by the Fragment Analyzer™ in the eluate of the QIAamp DNA Blood Mini Kit and the PCI together, ranging from ∼100 to ∼700 bp, might be present in the plasma samples, with each DNA purification procedure only yielding certain fractions. A trial to analyze the cfDNA fragment lengths directly in plasma failed. The plasma sample was implemented in a 1∶40 dilution in H_2_O in the Fragment Analyzer™ measurement (data not shown).

The DNA integrity was lower post exercise compared to the point in time pre exercise in unpurified plasma and in the eluate of the QIAamp DNA Blood Mini Kit and the PCI. Our qPCR data indicated that the determination of DNA integrity values strongly depended on the sample treatment. These findings were confirmed by the outcomes of the Fragment Analyzer™ measurements, showing different fractions of fragment lengths for the same plasma samples. In addition, the published studies about DNA integrity values in healthy and patient collectives are hardly comparable because different amplicon lengths were analyzed, respectively [22], [24], [37], [49], [50]. The analyses of cfDNA concentrations or integrity indices are of great importance for clinical diagnostics and therefore, it is imperative to define standard procedures. These standard procedures should comprise direct application of unpurified plasma or serum for the measurement of the veritable cfDNA content in the sample, and the amplification of two assigned sequence lengths in the qPCR.

The mean cfDNA concentrations measured in the plasma of CHD patients were 2-fold higher compared to healthy controls, although no acute medical conditions were present. CHD is characterized by chronic arteriosclerotic narrowing of the coronary vessels, leading to oxidative stress and myocardial ischemia. Occurring arteriosclerotic lesions lead to inflammatory processes with subsequent release of reactive oxygen species from macrophages [31]. Acute events in the progression of CHD like ACS occur when the impairment of myocardial perfusion exceeds a critical threshold, leading to acute ischemic conditions that require immediate medical care [51]. Cui et al. [31] stated that cfDNA concentrations in patients with stable angina were lower compared to patients suffering from ACS. Therefore, cfDNA concentrations in the plasma of CHD patients might mirror the present condition, with increasing cfDNA concentrations the more ischemic or inflammatory processes occurred inside the organism. There have been several studies describing the association of cfDNA levels with the severity of different clinical conditions [34], [52], [53] that might support this hypothesis. Until this association can be scientifically assessed, future investigations need to manifest a cfDNA-associated (epigenetic) measure with specificity to myocardial perfusion. Like it has been recommended for the assessment of ACS [54], further studies also have to clarify, if elevated cfDNA levels in the plasma of the CHD patients result from this syndrome or from other comorbidities like hypertension or hypercholesterolemia [55]. Since the control subjects in our module were significantly younger and slighter than the CHD patients, we cannot exclude that factors like age and weight had impact on the cfDNA concentrations. However, with regard to the age and associated body dimensions, the collective of healthy controls represented the average German population (www.gbe-bund.de).

Both, the patients and the healthy controls in Module 2 yielded lower mean baseline cfDNA concentrations than the sportive subjects in Module 1. This might be due to the different sampling sites or due to the fact that the athletes in Module 1 took part at an official relay race, where there was no chance to control their warming-up before sampling. Furthermore, their exercise schedules during the days before the race remained unknown and thus these subjects might have exhibited chronically elevated cfDNA baseline levels due to repeatedly high training loads [6].

In conclusion, the phenomenon of pathological and exercise-induced increases of cfDNA remains widely unexplained and requires further investigations *in vivo* and *in vitro*. As research potentialities in clinical purposes are limited, the best way to monitor cfDNA release mechanisms *in vivo* is given by physical exercise. It provides the opportunity to deliberately induce and continuously observe cfDNA variations [7]. Possible triggers might be given by exercise intensity and duration as a result of oxidative, metabolic or mechanical stress [8]. However, it should be clarified first, if exercise-induced cfDNA accumulations can be transferred to pathological conditions in terms of origin and release mechanism.

With our new direct qPCR, we recommend a simple, fast and sensitive procedure for the absolute quantification of cfDNA concentrations in diverse fluids without preceding DNA purification. This procedure is considerably more time effective, reveals significantly higher cfDNA concentrations compared to different DNA extraction methods and preserves from the loss of fractions of fragment lengths, or rupture or adhesion of cfDNA fragments during DNA extraction. However, like it has been established for every method measuring cfDNA, this procedure still affords the centrifugation of whole blood to remove blood cells and their nuclear DNA that might already induce loss of cfDNA fragments bound to bigger molecules. Subsequent investigations should clarify the suitability of centrifugation to reliably determine physiological cfDNA concentrations. Nevertheless, our direct L1PA2 qPCR is applicable for diverse *in vivo* and *in vitro* settings and will provide progress on our way to understanding the phenomenon of cfDNA.

## Supporting Information

Methods S1 This section describes the DNA extraction using phenol-chloroform isoamyl (PCI), the MSTN qPCR and the quantification of cfDNA in the flow-through of the spin columns of the QIAamp DNA Blood Mini Kit.
(DOCX)
