# Supplementary material for: Direct Quantification of Cell-Free, Circulating DNA from Unpurified Plasma
Source: PLoS One. 2014 Mar 3;9(3):e87838. doi: 10.1371/journal.pone.0087838 (PMC3940427; doi:10.1371/journal.pone.0087838)
Supplement: Methods S1 — This section describes the DNA extraction using phenol-chloroform isoamyl (PCI), the MSTN qPCR and the quantification of cfDNA in the flow-through of the spin columns of the QIAamp DNA Blood Mini Kit. (DOCX) [file pone.0087838.s001.docx]

Methods S1

Phenol-chloroform isoamyl extraction

100 µl of plasma were added to 500 µl PBS and 6 µl Triton-X (Carl Roth GmbH + Co KG, Karlsruhe, Germany) and vortexed. The mixture was incubated for 5 minutes at 98°C for protein denaturation and then cooled down on ice for 5 minutes. After addition of 600 µl phenol-chloroform isoamyl (PCI; Sigma-Aldrich Co., Taufkirchen, Germany), the samples were vortexed for 30 seconds and centrifuged for 10 minutes at 16,000*g and room temperature. The aqueous phase was then transferred to a fresh tube and mixed with 1400 µl ethanol 100% and 60 µl 3 M sodium acetate. After precipitation over night at -20 °C, the DNA was pelleted by centrifugation for 30 minutes at 16,000*g and 4°C. The supernatant was removed and discarded. Three washing steps were performed with 1000 µl ethanol 70% in steps one and two and 1000 µl ethanol 100% in step three and centrifuged for 5 minutes at 16,000*g and 4°C, respectively. After the removal of the supernatant of the third washing step, the samples were incubated in an open tube at 55°C until the whole ethanol was evaporated. Finally, the purified cfDNA was eluted in 20 µl TE buffer and stored at -20°C until measurement.

MSTN qPCR

For the amplification of an 88 bp fragment of the human myostatin (MSTN) gene, cfDNA concentrations were quantified by SYBR green real-time PCR using the primers 5’-TTGGCTCAAACAACCTGAATCC-3’ and 5’-TCCTGGGAAGGTTACAGCAAG-3’. qPCR measurements were carried out on a MyiQ single-color detection system (BioRad, München, Germany). The thermal cycler protocol started with 95 °C for 5 min followed by 40 cycles of a three-step reaction: denaturation at 94 °C for 15 s, annealing at 59 °C for 30 s, and extension at 72 °C for 10 s. Each single reaction included 5 µl of plasma eluate, 7.5 µl of QuantiFast SYBR Green PCR Kit (Qiagen GmbH, Hilden, Germany) and primers (0.3 µM), resulting in a final volume of 15 µl per reaction. Samples were run in triplicates. More detailed information may be obtained from Beiter et al. [7].

Quantification of cfDNA in flow-through of isolation with spin columns

The flow-through of all washing and centrifugation steps of the QIAamp DNA Blood Mini Kit (Qiagen GmbH, Hilden, Germany) was collected in a tube for each sample. A 1:40 dilution of the flow-through in H_2_O was measured according to the introduced L1PA2 qPCR protocol for the quantification of cfDNA losses during the DNA isolation. To investigate the possible inhibitory effect of ethanol, proteinase K or high salt concentrations in the flow-through on the qPCR efficiency, the L1PA2 standard dilution series was measured with both L1PA2 primers (for 90 and 222 bp amplicons) in a master mix containing an equivalent proportion of buffers and ethanol. The standard curve showed equal axis and slope compared the standard curve without background noise (pure standard curve in TE buffer). Therefore, the qPCR efficiency was not affected by the mixture of buffers and ethanol.
